# Unveiling the Pharmacological and Nanotechnological Facets of Daidzein: Present State-of-the-Art and Future Perspectives

**DOI:** 10.3390/molecules28041765

**Published:** 2023-02-13

**Authors:** Sukhbir Singh, Sonam Grewal, Neelam Sharma, Tapan Behl, Sumeet Gupta, Md. Khalid Anwer, Celia Vargas-De-La-Cruz, Syam Mohan, Simona Gabriela Bungau, Adrian Bumbu

**Affiliations:** 1 Department of Pharmaceutics, MM College of Pharmacy, Maharishi Markandeshwar (Deemed to Be University), Ambala 133207, India; 2 School of Health Sciences & Technology, University of Petroleum and Energy Studies, Bidholi, Dehradun 248007, India; 3 Department of Pharmacology, MM College of Pharmacy, Maharishi Markandeshwar (Deemed to Be University), Ambala 133207, India; 4 Department of Pharmaceutics, College of Pharmacy, Prince Sattam Bin Abdulaziz University, Alkharj 11942, Saudi Arabia; 5 Department of Pharmacology, Bromatology and Toxicology, Faculty of Pharmacy and Biochemistry, Universidad Nacional Mayor de San Marcos, Lima 15081, Peru; 6 E-Health Research Center, Universidad de Ciencias y Humanidades, Lima 15081, Peru; 7 Substance Abuse and Toxicology Research Centre, Jazan University, Jazan 45142, Saudi Arabia; 8 Center for Transdisciplinary Research, Department of Pharmacology, Saveetha Dental College, Saveetha Institute of Medical and Technical Science, Saveetha University, Chennai 602117, India; 9 Department of Pharmacy, Faculty of Medicine and Pharmacy, University of Oradea, 410028 Oradea, Romania; 10 Doctoral School of Biomedical Sciences, University of Oradea, 410087 Oradea, Romania; 11 Department of Surgery, Faculty of Medicine and Pharmacy, University of Oradea, 410073 Oradea, Romania

**Keywords:** daidzein, nanotechnology, polymeric nanoparticles, pharmacological applications, solid lipid nanoparticles, polymer-lipid nanoparticles

## Abstract

Herbal drugs have been attracting much scientific interest in the last few decades and nowadays, phytoconstituents-based research is in progress to disclose their unidentified medicinal potential. Daidzein (DAI) is the natural phytoestrogen isoflavone derived primarily from leguminous plants, such as the soybean and mung bean, and its IUPAC name is 4′,7-dihydroxyisoflavone. This compound has received great attention as a fascinating pharmacophore with remarkable potential for the therapeutic management of several diseases. Certain pharmacokinetic properties of DAI such as less aqueous solubility, low permeability, and poor bioavailability are major obstacles restricting the therapeutic applications. In this review, distinctive physicochemical characteristics and pharmacokinetics of DAI has been elucidated. The pharmacological applications in treatment of several disorders like oxidative stress, cancer, obesity, cardiovascular, neuroprotective, diabetes, ovariectomy, anxiety, and inflammation with their mechanism of action are explained. Furthermore, this review article comprehensively focuses to provide up-to-date information about nanotechnology-based formulations which have been investigated for DAI in preceding years which includes polymeric nanoparticles, solid lipid nanoparticles, nanostructured lipid carrier, polymer-lipid nanoparticles, nanocomplexes, polymeric micelles, nanoemulsion, nanosuspension, liposomes, and self-microemulsifying drug delivery systems.

## 1. Introduction

Therapies based on compounds derived from plants that grow in nature have always been a symbol of the extraordinary phenomenon of symbiosis in the body. Moreover, herbal medicines have their ancestry in every culture all over the world [1,2]. The severe adverse effects of allopathic treatments on individual health have encouraged the emergence of natural remedies as potential therapeutics for acute and chronic disorders. The thousands of medicinal plants are utilized around the planet, which is attributed to their contribution towards potent therapeutic agents with numerous potential therapeutic effects, without producing serious side effects and, therefore, herbal medicines, by implicating plant-based compounds, have been accepted as being useful a remedy for numerous diseases [3,4,5].

Additionally, medicinal plants offer a rich source of beneficial phytoconstituents that could be investigated for the development of novel drug delivery systems [6]. Alkaloids, tannins, flavonoids, and phenolic compounds are significant bioactive plant components which play an explicit role in the mitigation of a range of health issues as well as chronic diseases [7,8]. The investigations of secondary plant products have progressed comprehensively in the last few decades. Flavonoids are a group of secondary plant metabolites with polyphenolic structure and are generally found in fruits, vegetables, and certain drinks [9]. Flavonoids are classified into subgroups such as catechins, anthocyanins, chalcones, flavones, flavonols, flavanones, flavanonols, and flavanols [10,11]. Numerous studies have revealed that flavonoids have potential to avert several bacterial and viral infections [12], cancer [13], arthritis [14], osteoporosis [15], diabetes [16], skin disorders [17], cardiovascular disease [18], and other age-related illnesses [19].

Daidzein (DAI) belongs to the isoflavone class of flavonoids which are commonly consumed by Western populations such as North Americans and Europeans in relatively modest amounts and in relatively high concentrations by Asian populations such as the Chinese and Japanese [20]. DAI is a type of naturally occurring, non-steroidal isoflavone that is typically derived from leguminous plants such as the soybean and mung bean [21]. Food products from soy, such as tofu, tempeh, miso, textured soy protein, soy flour, and soy protein isolates, contain DAI. The amount of DAI in a cup of soy milk is 7 mg, a half-cup of miso is 22 mg, three ounces of tempeh is 15 mg, and three ounces of tofu is 8 mg [22].

The physicochemical properties and pharmacokinetics profile of DAI has been revealed in the current review. This article summarizes the pharmacological applications and mechanisms of action of DAI in the management of many disease conditions, including oxidative stress, cancer, obesity, cardiovascular, neuroprotective, diabetes, ovariectomy, anxiety, and inflammation. In addition, the objective of the current review article is to provide up-to-date knowledge on the nanotechnology-based approaches investigated in the past to increase the solubility and permeability of DAI. These approaches include polymeric nanoparticles, solid lipid nanoparticles, nanostructured lipid carriers, polymer-lipid nanoparticles, nanocomplexes, polymeric micelles, nanoemulsion, nanosuspension, liposomes, and self-microemulsifying drug delivery system. For this purpose, a comprehensive literature survey was conducted using the Google Scholar, PubMed, and ScienceDirect databases. The research and review papers published in peer-reviewed journals between the years 2000 and 2022 served as the basis for the literature review.

## 2. Physicochemical Properties and Pharmacokinetics Profile of Daidzein

DAI has the IUPAC name 4′, 7-dihydroxylisoflavone and is a water-insoluble isoflavone, existing as pale yellow crystalline prisms and having a partition coefficient of 2.55; its solubility in the different solvents was in the sequence like propanone > methanol > ethyl ethanoate > hexane > trichloromethane > water [23], and in aqueous buffer (with pH 6.0) was found 18.76 nmol/mL [24].

Some of DAI’s unfavorable physicochemical characteristics (poor solubility, low partition coefficient, and high intestine and hepatic metabolism) lead to low oral bioavailability. According to animal model studies, absolute bioavailability of DAI suspension after oral administration to rats was 6.1%, which seems to be the greatest constraint limiting therapeutic and pharmacological uses. There have been numerous approaches investigated to make DAI more bioavailable by derivatizing ionizable groups (such as sulfation, phosphating, or glycosylation of DAI) to more water-soluble forms [25,26,27,28,29,30].

DAI was administered to healthy premenopausal women, and the results showed that it has a low bioavailability and non-linear pharmacokinetics with higher intakes, showing that its absorption is rate-limited and saturated [31,32].

Intestinal microbiota has a significant effect on the metabolism and bioavailability of isoflavones, and it has been discovered that isoflavones cannot be absorbed without microbiota [33]. The bioavailability and absorption of isoflavones may be influenced by the bacterial flora of the stomach. Some isoflavones are ingested in their chemically modified form because the stomach may convert relatively weak molecules into stronger forms. DAI can be converted by intestinal microbiota into a number of substances, such as odesmethylangolensin, dihydrodaidzein, and 7-hydroxyisoflavan [34].

The blood–brain barrier allows daizein-8-C-apiosyl-(1-6)-glycoside to enter the brain quickly, and it may be detected in the brain within an hour of administration [35,36].

The diet of the human population is largely composed of soy products. As opposed to less than 2 mg in Western countries, the Asian population can consume up to 50 mg of isoflavones per day, although this number may be higher in menopausal women [20].

DAI has a 336.25 L volume of distribution, a 30.09 L/h clearance rate, and a 7.75 H half-life, respectively [37,38]. Absolute and relative bioavailability of DAI suspension (20 mg/kg i.v. vs. 50 mg/kg i.p.) and complexed form (0.54 mg/kg i.v. vs. 1.35 mg/kg i.p.) were evaluated. DAI complexed was absorbed more quickly (tmax = 15 min) and to a greater extent (Cmax = 615 vs. 173 ng/mL) following intraperitoneal administration than DAI in suspension (tmax = 45 min). DAI’s i.v. half-life was longer in the complex of DAI when compared to DAI in suspension (t_0.5_ = 80 min vs. 230 min) [31].

**Figure 1:**
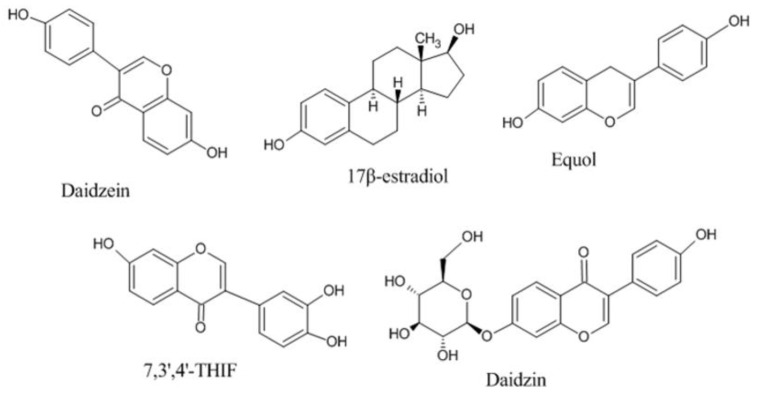
Chemical structures of Daidezin and its various analogs.

The physicochemical characteristics and pharmacokinetic profile of DAI have been summarized in Table 1 and Figure 1 depicts the chemical structure of DAI and its various analogs [21].

## 3. Mechanism of Action and Pharmacological Applications of Daidzein

Numerous pharmacological effects of DAI include anti-carcinogenesis [42], anti-inflammatory [43], antioxidant [44], anti-diabetic [45], cholesterol-lowering [46], and cardiovascular activity [47,48].

DAI imitates human estrogen, which has a substantial impact on the prevention of osteoporosis, cancer, and postmenopausal disorders. Soy products are highly recommended for cancer prevention due to high content of anticarcinogens in them [49,50,51].

Matrix metalloproteinase-2 activity is inhibited by DAI to produce an anticancer effect, and its non-toxic concentration is also extensively used to modulate Hedgehog signaling to prevent tumor necrosis factor-induced migration and the invasion of human breast cancer cells [52].

DAI significantly raises high density lipoprotein cholesterol (HDL-C) levels, lowers levels of circulating triglycerides (TGs) and low density lipoprotein cholesterol (LDL-C), and thus, prevents heart attack or stroke [53]. Additionally, it increases the expression of bone morphogenetic protein (BMP) in primary osteoblast cells, promoting the development of osteoblast, which ultimately exhibited anti-osteoporosis activity [54]. Moreover, DAI increases the ratio of glucose transporter-4 (GLUT4) to Na^+^/K^+^ ATPase levels, which facilitate in glucose absorption and maintain the proper balance of reactive oxygen species to free radicals [55,56].

**Figure 2:**
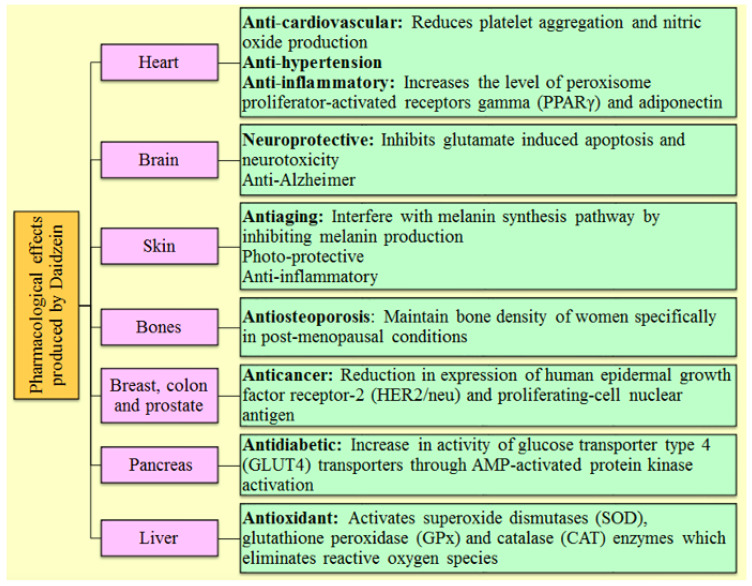
Representation of several pharmacological activities of Daidzein and their mechanism of action involved in various body organs.

Different analogs of DAI (such as equol, 17 β-estradiol, 7, 3′, 4′-THIf and daidzin) exhibited the similar mechanism as DAI by binding with the protein kinase B, estrogen receptors, mitogen-activated protein kinase, and epidermal growth factor receptor kinase, nuclear factor kappa-light-chain-enhancer of activated B cells, and other intracellular signaling mechanisms [30,57]. Figure 2 illustrates the numerous mechanisms through which DAI exerts its therapeutic potential in a variety of essential body organs.

### 3.1. Anticancer Activity

Polycyclic phenolic phytochemicals known as phytoestrogens have characteristic structures that resemble steroidal estrogen. Their ability to treat and prevent cancer has recently received a lot of attention. Increased consumption of foods and herbal remedies containing phytoestrogens plays a crucial part in lowering estrogen levels and the prevalence of breast cancer [58,59].

By reducing the activity of matrix metalloproteinase-2, DAI prevented the MDA-MB-231 breast cancer cell lines from attacking them, indicating a significant function for DAI in the development of breast cancer [52,60].

TNF-induced nuclear localization of the gene glioma-associated oncogene homologue-1 (Gli1) and genetic expressions into mRNA and protein that inhibited TNF-induced migration and invasion in human breast cancer cells have been studied extensively using DAI to control Hh-signaling [52,61]. DAI inhibited the proliferation of cell lines originated from cancer, and as a result, apoptosis was induced in cancer cells. Depending on the kind of cancer cell, it can be used to increase apoptosis linked to G0/G1 cell cycle arrest. Direct apoptosis is caused by the S- or G2-phase without altering cell distribution [62].

The impact of DAI’s antiproliferative properties on human breast cancer cell lines, i.e., MCF-7 and MDA-MB-453, at dosages ranging from 1 to 100 mM for 24, 48, and 72 h, showed reduced cell proliferation in both types of cells in a dose- and time-dependent manner [63].

Wang et al. demonstrated that 145 mg/kg of DAI administered orally for 22 days causes breast cancer cells to undergo apoptosis via the Fas/FasL-initiated mitochondrial apoptosis signaling pathway in bearing-4T1 mice [64].

Numerous studies have demonstrated that DAI has therapeutic advantages for the treatment of malignancies other than breast cancer. Moreover, it demonstrated anti-proliferative activities in three prostate cancer cell lines (DU 145, LNCaP, and PC-3), modulating the gene expression associated with the cyclin-dependent kinase-related pathway, resulting in cell cycle arrest at the G0/G1 phase, and suppressing angiogenesis. A few of these genes are involved in the angiogenesis process and the DNA damage signaling mechanism, which can lower levels of the epidermal growth factor and insulin-like growth factor and therefore prevent the development of tumors [65].

LoVo cells displayed a tumor-suppressing impact as a consequence of cell cycle arrest at the G0/G1 phase and caspase-3-dependent apoptosis, which had no effect on differentiation. In numerous murine as well as human neuroblastoma cell lines, DAI exhibited its anticancer potential by inhibiting cell growth, arresting the cell cycle during the G2/M phase, and promoting cell death [66].

Due to the biotransformation of DAI, this can be utilized as a chemo-preventive drug in skin cancer despite its lack of effect on cyclooxygenase 2 (COX-2) expression, its metabolite directly binding to tumor progression locus and mitogen-activated protein kinase 4 to block their activity. This significantly lowers the ultraviolet B-induced COX-2 expression and, subsequently, prevents tumor growth, development, and enlargement [57].

### 3.2. Cardiovascular Diseases

While postmenopausal women have a greater incidence of cardiovascular disorders than premenopausal women [67], males aged 35 to 50 had a higher incidence of cardiovascular diseases than women of equivalent ages [68].

The endothelium’s ability to produce nitric oxide is activated by estrogen receptors, and blood vessels are also relaxed by prostacyclin and hyperpolarizing factor. It is possible that using natural phytoestrogens in small doses has advantages over using synthetic estrogen [69]. Low levels of HDL-C, as well as high levels of TGs and LDL-C, are important risk factors for cardiovascular disease. Six months of DAI therapy in hypercholesterolemic patients can considerably lower triglyceride and uric acid levels in blood, but not in a dose-dependent way [53].

Caveolin, a transmembrane protein, is present in the minute caveolae that project from the plasma membrane. Caveolin-1, a specific marker of caveolae, tends to up-regulate expression in response to conditions such elevated levels of oxidized low density lipoprotein, estrogen deficiency, and hyperglycemia [70]. It functions as a protein that binds to cholesterol and makes it easier for cholesterol to go from the endoplasmic reticulum to the plasma membrane’s endothelial cells via the Golgi apparatus. DAI functions as a caveolin-1 inhibitor, which has the potential to raise endothelial nitric-oxide synthase (eNOS) activity and to improve the vascular endothelium due to an increase in nitric oxide generation and stimulation of eNOS through caveolin-1 inhibition [71].

### 3.3. Anti-Osteoporosis Activity

Menopause causes the condition of equilibrium in the body to shift in favor of greater resorption, which lowers the bone mineral density and disturbs the bone microarchitecture [72]. The metabolism of bones and the growth of bone mass are influenced by systemic hormones, genetics, and environmental factors [73].

According to the conventional view, osteoporosis is a “breakable bone” disorder that primarily affects post-menopausal Caucasian women and those who consume insufficient levels of calcium and vitamin D [74].

DAI has received the most scientific attention among soy phytoestrogens, and numerous studies have demonstrated that it may have antiosteoporosis potential. DAI stimulates osteoblast formation in mouse osteoblast-like MC3T3-E1 cells via increasing BMP expression in primary osteoblast cells, which in turn promotes cell differentiation and mineralization [75].

DAI treatment prevents bone mass loss in both juvenile and adult ovariectomized rats and appears to promote protein synthesis and alkaline phosphatase in bone development. Phosphatase mineralization, which has been examined after being cultivated in osteoblast-like MC3T3-E1 cells, is an indication of osteoblast-induced matrix maturation [76]. Additionally, DAI greatly increases the activity of alkaline phosphatase, sodium-deoxyribonucleic acid, and calcium in bone tissue [54].

### 3.4. Antidiabetic Activity

Diabetes is currently posing a challenge upon India because it is progressively acquiring the position of a possible epidemic [77]. The significant mortality and cardiovascular morbidity of diabetes patients also contributes to the rise in demand for bio compounds with antidiabetic characteristics [78].

DAI inhibits the rise in blood glucose levels and promotes glucose absorption in adipocytes and muscle cells. Additionally, it increases the ratio of GLUT4 to Na^+^/K^+^ ATPase in the plasma membrane portion of L6 myotubes, indicating that this phytoconstituent may promote glucose absorption by GLUT4 translocation from intracellular micro vesicles [56,79].

DAI has a lower risk of hypoglycemia due to its minimal effect on insulin production and lack of influence on fasting blood sugar levels, significantly decreasing blood sugar levels and raising oral glucose tolerance when administered orally to diabetic mice, which had a significant impact on hyperglycemia. It evidently reduces blood levels of total cholesterol, triglycerides, and LDL-c while modestly raising blood levels of HDL-c. Therefore, this was disclosed that oral administration of DAI is effective in treating hyperglycemia and diabetes-related disorders [80].

### 3.5. Antioxidant Activity

Soybeans contain large amounts of the isoflavone DAI and is consumed in enormous quantity by Asian populations. Isoflavones have been associated with beneficial health effects as a result of their antioxidant properties due to their ability to cause chelation of toxic metal ions [81].

Dietary DAI is frequently transformed by intestinal bacteria into substances like 3′-OH-daidzein and 6′-OH-daidzein, which have powerful antioxidant potential compared to the parent molecule DAI. The antioxidant effects of DAI-induced antioxidant benefits may be mediated by DAI metabolites generated in the gut [82].

The potential of DAI to chelate copper ions results in its antioxidant activity. The Cu^2+^ has a propensity to stimulate lipoprotein oxidation in serum, which causes the LDL particles to aggregate and fuse. The chelation of Cu^2+^ has an antioxidant effect and protects against the oxidative transformation of LDL [83].

### 3.6. Anti-Inflammatory Activity

Inflammation is a biological response triggered upon by infections, damaged cells, and irritants [84]. Anti-inflammatory drugs, whether steroidal or nonsteroidal, are frequently used to treat inflammation, but they frequently have several adverse side effects. Recent studies have demonstrated that polyphenols derived from plants, in particular flavonoids, have potent anti-inflammatory activities [85].

Chronic/acute intestinal inflammation are both correlated with abnormal mucosal immune responses. Inflammatory bowel disease and increased pro-inflammatory chemical production are typically the two main pathogenic factors in chronic inflammatory diseases [86].

An imbalance between the synthesis of reactive oxygen species and antioxidant activity is known as oxidative stress which causes tissue damage. DAI 100 μM decreased interleukin-1β, interleukin-6, and tumor necrosis factor-α expression by 73.8 ± 5.3%, 58.8 ± 9.0% and 55.5 ± 7.2%, respectively. Through the downregulation of Kelch-like ECH-associated protein 1 and the upregulation of nuclear factor erythroid 2-related factor 2 expression, it also decreased the formation of reactive oxygen species caused by lipopolysaccharide by 23.9 ± 7.8% and enhanced superoxide dismutase activity by 88.4 ± 18.9% [43]. Oxidative stress is a condition that is often brought on by an increase in free radicals and reactive oxygen species [55,87].

In order to prevent human diseases and maintain proper health conditions by avoiding oxidative stress, an increase in antioxidant intake is required. DAI’s gut microbial metabolites O-desmethylangolensin (O-DMA), equol, and daidzin have antioxidant properties in the following sequence: DAI > equol > O-DMA > daidzin [88].

### 3.7. Neuroprotective Activity

DAI can prevent the progression of neurodegenerative diseases. Beta-secretase and cholinesterase are scientifically identified targets of Alzheimer’s disease, and both have benefited significantly from bio compounds. Given that Alzheimer’s disease is a serious public health issue, it requires the use of multiple-targeted drugs to be treated [89].

Stroke has a high morbidity rate globally, and there are currently no viable treatments for this disease [90]. Strokes are known to be associated with brain damage that permanently harms the body, while DAI aids in neuroprotection and functional recovery following a stroke [91].

DAI has neuroprotective effects in stroke conditions and has shown peroxisome proliferator-activated receptor gamma (PPAR-γ)-dependent therapeutic effects in brain cells and has huge potential to improve synaptic functioning in cultured neurons. An experimental study found that DAI increased PPAR-γ transcriptional activity while suppressing selective PPAR-γ antagonist [92].

In ischemic, neurodegenerative, and inflammatory brain disorders, PPAR activity assists in preventing neuronal death [93]. DAI treatment produced an anxiolytic effect in treated males by significantly increasing locomotor activity, improving harmonious behavior, reducing hostility, and reducing sexual behavior during social interaction [94].

Table 2 provides a systematic summary of pre-clinical investigations carried over the past few decades exploring the pharmacological applications of DAI in conditions such as oxidative stress, cancer, obesity, cardiovascular, neuroprotective, diabetes, ovariectomy, anxiety, and inflammation. It also includes information on animal models used and study outcomes.

## 4. Outline of Nanotechnological Aspects Explored for Daidzein in Therapeutics

DAI has limited clinical applications because of poor aqueous solubility and less permeability which causes low oral bioavailability. In the light of available information, the development of nanoparticles is a suitable strategy to address issues of low solubility, permeability, and bioavailability.

A significant role of nanomedicine in the treatment of many disorders has been demonstrated in research conducted in this field. Utilizing nanotechnology enables early diagnosis and more effective drug administration. Nanomaterials range in diameters between 1 and 1000 nm and have a large surface area to volume ratio. According to their structural properties, nanomaterials can be classified as either nanostructured or nanocrystalline. Nanostructured materials can be divided into three categories: lipid-based, polymer-based, and non-polymer-based depending on the type of material used [115,116,117].

The numerous applications of nanotechnology in the pharmaceutical sector have been demonstrated in areas such as targeted diagnostics, therapy, delaying drug release, enhancing drug solubility and bioavailability, reducing drug adverse effects, and overcoming barriers in the human body [118].

Table 3 summarizes the recent advancements in the field of nanotechnology-based drug delivery systems of DAI which has been investigated to improve solubility and bioavailability (Table 3).

There are several methods for encapsulating DAI nanoparticles, including the solvent evaporation method [26], antisolvent method [119], emulsion solvent diffusion [120], hot homogenization [121], film homogenization [29], media milling [27], ultrasonication/lipid film hydration method [129], and emulsification [123].

The experimental study showed that poly(lactic-co-glycolic acid) (PLGA) and PLGA-Gelucire nanoparticles loaded with DAI were used to treat glioblastoma multiforme, and it led to the conclusion that the formulation used was effective for sustained delivery, reducing neurotoxic effects, and maintaining cytotoxic effects against cancer cells [120].

DAI is a very useful medication for the treatment of cardio-cerebrovascular illnesses, but it is not as effective as it might be because of its poor oral absorption and bioavailability. A group of researchers prepared solid lipid nanoparticles for treatment of cardio-cerebrovascular diseases. The prepared solid lipid nanoparticles released the drug in a sustained manner and demonstrated over 90% release within 120 h [121].

**Figure 3:**
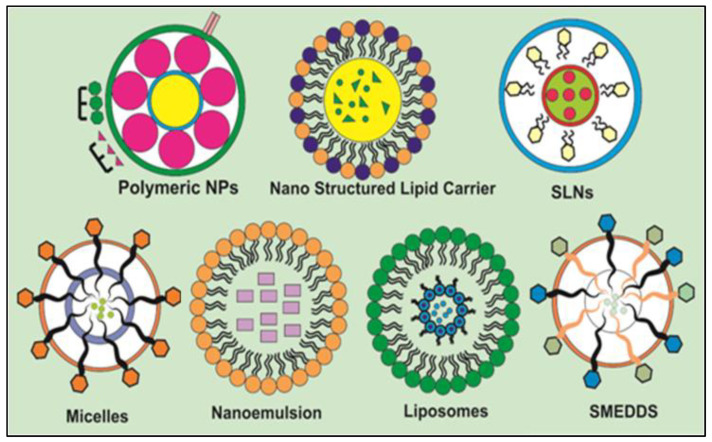
Structural composition of various nanocarriers explored for encapsulation of DAI regarding solubility and bioavailability enhancement.

The structural composition of several nanocarriers investigated for innovative delivery of DAI is shown in Figure 3.

### 4.1. Polymeric Nanoparticles

Polymeric nanoparticles (PNPs) are a type of particle with sizes ranging from 1 to 1000 nm, that are comprised of active compounds that have been entrapped inside the polymeric core or surface-adsorbed onto the polymeric core [130].

PNPs’ ability to protect drugs and their potential for controlled release can increase drugs’ bioavailability and therapeutic index [131]. PNPs, which contain a variety of therapeutic compounds, are produced with biodegradable materials such as poly-(D, L-lactic acid), PLGA, polycaprolactone, and its copolymers such as polyethylene glycol [132].

PNPs can be synthesized from two methods, i.e., (i) dispersion of performed polymers and (ii) polymerization of monomers. Dialysis, nanoprecipitation, solvent evaporation, supercritical fluid technology, emulsification, solvent diffusion, and salting out are the methods utilized to disperse the performed polymers. Another method for producing PNPs using microemulsion polymerization, controlled radical polymerization, and interfacial polymerization involves the polymerization of monomers [133,134,135].

A group of researchers formulated DAI PLGA nanoparticles using the emulsion-solvent evaporation method, and relative bioavailability was enhanced about 5.57- and 8.85-fold, respectively, in comparison to the control group [26]. By employing the anti-solvent approach, Zou and Gu synthesized TPGS 1000 emulsified zein nanoparticles, and they discovered that nanoparticles had increased Cmax of DAI by 2.64-fold and are under the curve (AUC) (0–12 h) by 2.4-fold compared to free drug [119].

### 4.2. Solid Lipid Nanoparticles

Solid lipid nanoparticles (SLNs) are efficient colloidal carriers which have fascinating characteristics such as small size, large surface zone, high drug entrapment, and the capacity to improve the therapeutic performance of pharmaceuticals [136,137].

Aqueous surfactant is coated over a solid core of high melting point lipid in SLNs. Triglycerides, acyl glycerol, glyceryl monostearate, waxes, cetyl palmitate, soy lecithin, and egg lecithin are among the several lipid types employed in the production of SLNs [138,139].

A number of techniques are employed to prepare SLNs, including double emulsion (w/o/w), ultrasound dispersion, high shear homogenization, solvent emulsification-diffusion, solvent injection, and high pressure homogenization (cold and hot homogenization) [140,141,142]. A group of researchers developed DAI solid lipid nanoparticles using hot homogenization method and it demonstrated sustained drug release with cumulative release over 90% within 120 h [121].

### 4.3. Nanostructured Lipid Carriers

Lipid-based formulations, such as nanostructured lipid carriers (NLCs), are regarded to be superior to conventional lipid-based nanocarriers, which have a rigid matrix at room temperature. NLCs are created by combining liquid lipid and solid lipid in such a way that prevents the oil molecules from contributing to the crystalline structure [143]. In order to overcome the drawbacks of SLNs, NLCs have been developed which demonstrated better loading capacity for active chemicals as compared to SLN. Moreover, there is less possibility of drug discharge from NLCs during storage [144].

The methods that are typically employed for the production of NLCs include film-ultrasonic, evaporation-low temperature solidification, high-pressure homogenization, microemulsion, supercritical fluid, membrane contactor, solvent dispersion, microchannel, and microtubes [145].

By using emulsification and low-temperature solidification technique, Song and his colleague produced DAI-loaded nanostructured lipid carriers for transdermal application. Researchers found that the permeation rate was 3.78 times higher than that of pure DAI solution [122].

### 4.4. Polymeric Micelles

Polymeric micelles are amphiphilic co-polymers that have formed into nanoscale colloidal particles with sizes between 5 and 100 nm above the critical micelle concentration. The aqueous media is used for the production of micellar core–shell structure in order to reduce hydrophobic segment’s interaction with the single chains of polymers [146].

Additionally, polymeric micelles have a unique core–shell structure with an inner core that serves as a nanocontainer for hydrophobic drugs and an outer shell that is surrounded by hydrophilic polymer shell. Numerous advantages of polymeric micelles include ease of production, efficient drug loading without chemical alteration of the parent molecule, and controlled drug release [147].

Researchers synthesized DAI micelles using the anti-solvent technique and demonstrated that the AUC0-t was 20 times higher than it was for the free drug [124].

### 4.5. Nanosuspension

The colloidal dispersion containing drug particles with a submicron size is known as nanosuspension. A pharmaceutical nanosuspension is comprised of colloidal biphasic particles that are stabilized by surfactants and polymers and are free of matrix components. According to research, nanosuspension boosts bioavailability and absorption, which results in a dose reduction for oral dosage forms [148,149].

Homogenization, wet milling, emulsification, solvent evaporation, precipitation or microprecipitation are common methods for manufacturing nanosuspensions [150,151].

The stability of the particles created by the nanosuspension depends on their size. When compared to other delivery methods, nanosuspensions have the benefit of being simpler and have the ability to overcome concerns with poorly lipid- and water-soluble compounds [152].

A group of researchers synthesized DAI nanosuspension by precipitation high-pressure homogenization and concluded that oral bioavailability increased by 1.63–2.19 times greater than that of crude DAI [125].

### 4.6. Nanoemulsion

Nanoemulsions are colloidal particle systems with submicron sizes (10–1000 nm) that serve as drug carriers. Solid spheres with an amorphous, lipophilic, and negatively charged surface constitute these carriers. These usually improve drug delivery systems by increasing the therapeutic potency of drugs and minimizing their adverse effects [153]. The primary applications of nanoemulsions include the treatment of reticuloendothelial system infections, liver enzyme replacement therapy, cancer treatment, and vaccination [154]. The phase inversion method, sonication method, and high pressure homogenization are the techniques utilized to create nanoemulsions [155].

Drugs that are poorly water soluble can have their bioavailability increased by using oil-in-water nanoemulsion. However, the difficulties in reducing droplet size and the requirement for specialized equipment and manufacturing procedures make the development of nanoemulsion an expensive operation [156].

Researchers formulated a nanoemulsion of DAI using high-pressure homogenization, and a study revealed that it significantly increased cell death as compared to pure DAI [127].

### 4.7. Liposomes

Liposomes are spherical, uni lamellar or multilamellar vesicles that are used to deliver drugs into cells through the cell membrane, which is made up of cholesterol and a phospholipid bilayer [157].

Hand shaking techniques, sonication techniques employing probe or bath sonicators, reverse phase evaporation techniques, and freeze dried rehydration techniques are all used to produce liposomes [158,159].

Liposomes are effective for intracellular delivery of deoxyribonucleic acid, ribosome, proteins, and peptides. Targeted drug delivery to diseased sites is facilitated by the long circulation residence times of liposomes. Compared to free complements, liposomal drugs are more efficacious and have reduced toxicities [160,161]. Researchers prepared DAI-loaded liposomes using ultrasonication and lipid film hydration and found that the t_1/2_, mean residence time_0-t_ and AUC_0-t_ of DAI in the liposomes were 1.8, 1.6, and 2.5 times higher than those in free DAI [128].

### 4.8. Self-Micro Emulsifying Drug Delivery System (SMEDDS)

The Self-Micro Emulsifying Drug Delivery System (SMEDDS) refers to isotropic compositions of synthetic or natural oils, liquid or solid surfactants, or hydrophilic solvents/co-solvents which possess the remarkable ability to generate fine oil-in-water (o/w) microemulsions on gentle agitation accompanied by dilution in an aqueous environment such as gastrointestinal fluids [162].

SMEDDS is a cutting-edge method for making lipophilic drugs more soluble in water, which eventually increases their bioavailability. SMEDDS is an ideal carrier that has great potential for producing drug delivery across intestinal aqueous boundary and consequently tends to improve the bioavailability because it can carry out drug delivery to the gastrointestinal tract (GIT) in the form of globules with sizes ranging from 1 to 100 nm and enormous specific surface area. Peptides that are susceptible to enzymatic hydrolysis may be transported to the GIT via SMEDDS. To obtain sustained drug release, polymer can be added to the SMEDDS formulation [163].

The main advantages that distinguish SMEDDS from other nanocarriers when compared to other drug delivery systems are its simplicity in manufacturing and scaling up. For large-scale production, SMEDDS requires relatively low-cost manufacturing equipment, such as a conventional mixer with agitator and volumetric liquid filling machinery [164].

Researchers synthesized SMEDDS of DAI using the emulsification process, and the results showed that the bioavailability was increased by about 2.5 times when compared to the control group [129].

## 5. Clinical Status of Daidzein

On the official website of ClinicalTrials.gov, a search was conducted for the clinical trials including DAI and its medicinal uses that have been completed to date. According to research, DAI has undergone four successful clinical studies. Table 4 summarizes study tile, sponsor condition, study type/allocation/intervention model, and number of clinical trials (NCT) [165].

## 6. Conclusions and Future Perspectives

DAI, an isoflavone flavonoid, has attracted a lot of attention in recent years due to its wide range of therapeutic benefits on oxidative stress, cancer, obesity, cardiovascular disease, neuroprotection, diabetes, ovariectomy, anxiety, and inflammation. Despite the wide range of biological activities that this phytoconstituent exhibits, there are certain limitations to DAI’s administration, including its poor water solubility, slow absorption, and limited oral bioavailability.

This review revealed a number of nanocarriers that have been investigated for the delivery of DAI, including polymeric nanoparticles, solid lipid nanoparticles, nanostructured lipid carriers, polymeric micelles, nanocomplexes, nanosuspension, nanoemulsion, liposomes, and self-micro emulsifying drug delivery systems. Additionally, our paper highlighted the results of several studies that focused into generating nanocarrier-based DAI to increase its pharmacological potential, and it ultimately showed that nanotechnology might be quite helpful in resolving solubility and permeability challenges faced by phytoconstituents in therapeutic applications. The use of soy products has expanded over the last few years due to DAI’s vital role in therapeutic applications.

On the other hand, a long-term high soy product diet could reduce the secretion of serum testosterone and, therefore, can cause complications in male fertility. Additionally, research is required to examine a novel extraction technique to produce DAI analogues with a greater bioavailability.

Nano formulations present a tremendous opportunity for investigating the effectiveness and bioavailability of DAI because of their small particle size, high specific surface area, increased surface reactivity, and superior adsorption capacity.

## Figures and Tables

**Table 1:** Description of physicochemical characteristics and pharmacokinetic profile of Daidzein (DAI).

Profile	Description
Physicochemical characteristics	
Source	In Soy products
Empirical Formula	C_15_H_10_O_4_
Molecular Weight	254.54 g/mol
Solubility	Sparingly soluble in aqueous buffers; soluble in organic solvents like ethanol, dimethyl sulfoxide and dimethyl formamide.
Partition coefficient	2.55
Physical appearance	Pale yellow prisms
Melting point	315 ± 5 °C
ƛ_max_ (Lambda maximum)	250 nm
Pharmacokinetics	
Absorption	Research showed that DAI exhibits passive, unsaturable transport absorption mechanism predominantly from distal part of small intestine of rats in comparison to proximal and medial parts. This was found that total DAI intestinal absorption was approximately 6% in 60 min [39]
Distribution	Volume of distribution: 336.25 L; Clearance rate: 30.09 L/h [37,38]
Metabolism	DAI is converted by the body to its aglycone form (without the glucose side chain) [40]; the main metabolite of DAI was found daidzein-7-O-glucuronide [25]
Excretion	Approximately, between 30 and 40 percent of DAI is excreted in urine [41]

**Table 2:** Recapitulation of the outcomes of DAI’s pharmacological studies in several pre-clinical investigations for various disease conditions.

Route	Disease	Dose/Duration	Outcomes	Animal Model	Ref.
i.p.	Oxidative stress	200 mg/kg for 2 days	Effective in reducing glutathione reserve, glutathione peroxidase activity and superoxide dismutase’s activity	Mice	[51]
i.p.	Inflammation	1, 5, 10 mg/kg once a day for 7 days	DAI produced significant anti mucosity activity at 10 mg/kg against 5-Fluorouracil induced mucositis	Mice	[95]
p.o.	Memory impairment	5 mg/kg	Administration of DAI acts on estrogen receptor to improve the memory loss condition	Mice	[96]
p.o.	Obesity	50 and 100 mg/kg for 30 consecutive days	Reduced the body and white adipose tissue weight of obese mice and ameliorated the hyperlipoidemia induced by high fat diet	Mice	[97]
p.o.	Parkinson	50, 100 mg/kg per day for 5 days	Significant improvement in neuronal degeneration in brain tissue	Rats	[98]
s.c.	Cardiovascular	200 mg/day for 7 days	Effective in enhancement of endothelial dependent relaxation	Rats	[99]
p.o.	Memory impairment	5 kg/mg	Improvement in the dysfunction due to scopolamine and enhanced learning capacity as compared to control group	Mice	[100]
p.o.	Diabetes	10 mg/kg	Potential antidiabetic activity showed via inhibitory effect on α-glucosidase and α-amylase	Mice	[101]
p.o.	Blood pressure	20 mg/kg for 2 weeks daily	Induced hypotensive and vasodilator effects by inhibiting Ca^2+^ influx	Rats	[102]
s.c.	Ovariectomy	0.2, 0.4 and 0.8 mg/kg per day for 1 week	Improved vascular endothelial dysfunction by inhibiting caveolin-1 and activation of PI3K-PKB/Akt pathway	Rats	[103]
p.o.	Diabetic retinopathy	25, 50, 100 mg/kg for 28 days	Prevented from the damage of retina in hyperglycemia condition by reducing oxidative stress	Rats	[45]
p.o.	Neuroprotective	2 or 20 mg/day for 4 weeks	DAI significantly decreased the concentration of malondialdehyde and act as pro-oxidant	Rats	[104]
p.o.	Anxiety	200 mg/kg	Long-term DAI ingestion produced considerable impact on social behavior, mood, and locomotion	Mice	[105]
i.p.	Inflammation	10 mg/kg/day	Significantly reduced the severity of L-arginine-induced acute pancreatitis while the anti-inflammatory and strong antioxidative properties are responsible for improvement	Rats	[106]
p.o.	Inflammation	1.0 g/kg chow for 12 weeks	Effective in decreasing MCP-1, TNF-α, and increased expression of adiponectin	Mice	[107]
p.o.	Immunomodulation	20 mg/kg biweekly	Significantly reduced IgG1 production, while increased the T-helper cells	Mice	[108]
p.o.	Neuroprotective	200 mg/kg for 15 days	Study showed neuroprotective effect when interacted with the receptor neurotensin1 and interleukin-10 pathways	Rats	[109]
i.p.	Obesity	50 mg/kg for 14 days	Significantly reduced body weight in rats and, as well, ameliorate the condition of hyperlipidemia, which can partially explain the anti-steatotic, cholesterol-lowering and insulin sensitizing effects	Rats	[110]
p.o.	Fatty liver	0.1 g per kg diet for 12 weeks	Effective in inhibiting the adiposity by the upregulation of genes involved in fatty acid β-oxidation and the anti-adipogenesis	Mice	[111]
p.o.	Diabetes	50 mg/kg for 4 weeks	Demonstrated that it is effective in decreasing blood glucose level and no effect on resistin level	Rats	[112]
p.o.	Diabetes	0.2 g/kg for 6 weeks	Effectively act as anti-hyperglycemic through the activation of glucokinase and inhibition of G6Pase, PEPCK, FAS, β-oxidation, and CPT in the liver	Mice	[113]
i.p.	Oxidative stress	100 mg/kg for 11 days	Effective in imparting protection against the nephrotoxic effect	Rats	[114]

p.o.: per oral; i.p.: intraperitoneal; s.c.: subcutaneous; PI3K-PKB/Akt: phosphatidylinositol-3-OH kinase/protein kinase B; MCP-1: Monocyte chemoattractant protein-1; TNF-α: tumor necrosis factor alpha; IgG1: Immunoglobulin G1; G6Pase: Glucose 6-phosphatase; PEPCK: Phosphoenolpyruvate carboxy kinase; FAS: death receptor involved in apoptosis expressed by insulin-producing beta cells; CPT: carnitine palmitoyl transferase.

**Table 3:** Review of up-to-date progression in development of nanocarriers based drug delivery of DAI for solubility and bioavailability enhancement.

Technique	Excipients	Study Outcomes	Ref.
Polymeric nanoparticles			
Solvent evaporation method	Poly (lactic-co-glycolic), Phosphatidylcholine, Hydroxypropyl-β-cyclodextrin	Relative bioavailability of phospholipid complex based PLGA nanoparticles improved by 5.57 while cyclodextrin complex based PLGA nanoparticles showed 8.85-fold enhancement of relative bioavailability in comparison to DAI suspension at dosage of 10 mg/kg in Sprague Dawley rats via p.o. administration	[26]
Antisolvent method	Zein, Coumarin-6, TPGS-1000	C_max_ and AUC_0–12h_ was increased by 2.64-fold and 2.4-fold, respectively as compared to daidzin solution on p.o. administration of zein nanoparticles in mice	[119]
Emulsion solvent diffusion method	PLGA, Polyvinyl alcohol	Nanoparticles exhibited sustained drug release. Neurotoxic effects at high dosages of DAI (200 µM and 300 µM) was decreased while maintaining cytotoxic effects on U87MG glioma cell lines	[120]
Solid lipid nanoparticles			
Hot homogenization method	Egg phosphatidylcholine, Compritol 800, polyethylene glycol, phosphatidylethanolamine	AUC0–∞ from i.v. administration of DAI NPs and free DAI was found 83.62 ± 1.89 µg·h/mL and 28.29 ± 1.29 µg·h/mL, respectively, which illustrated bioavailability enhancement in Sprague Dawley rats. SLNs (i.v.) exhibited superior result on CVS of Beagle dogs via reduction of myocardial oxygen consumption and coronary resistance in heart in contrast to DAI suspension (p.o.) or i.v. solution. SLNs also revealed superlative action on cerebrovascular system through enhancing cerebral blood flow and decreasing cerebrovascular resistance in Beagle dogs	[121]
Nanostructured lipid carriers			
Emulsification and low temperature solidification method	Azone, lecithin, Triethanolamine, Capric triglyceride, Tetrahydrofuran	NLC-nanofibers achieved high permeation of 21.71 μg/cm^2^ at 60 h using rat skin which was 3.78-folds greater than pure drug	[122]
Film homogenization technique	Glycerol monostearate, Sodium oleate, Soybean phospholipids	AUC_0-t_ from NLCs was increased by 6.87-times while from phospholipid complexes was enhanced 3.62-folds in comparison to pure DAI in rat model and therefore, NLCs were found effective nanocarriers to increase oral absorption of poorly absorbed lipophilic and hydrophilic compounds	[29]
Polymer-lipid nanoparticles			
Emulsification method	PLGA, egg lecithin, azone, tween 20	In vivo skin retention study using rat skin showed that steady state flux (Jss) from polymer-azone-lipid NPs was enhanced 1.44-folds and 6.01-folds in comparison to polymer-lipid-NPs and DAI solution	[123]
Micelles			
Solvent evaporation technique	Lecithin, sodium bile	Intestinal absorption of DAI from lecithin micelles was significantly improved in Sprague Dawley rats and AUC0-t value in rats receiving micelles treatment was twenty times higher than that of free DAI solution	[124]
Nanosuspension			
Precipitation-high pressure homogenization method	TPGS, carboxylated chitosan, Poloxamer 188, PVP-K30, Cremophor, PEG 600, β cyclodextrin, Soy lecithin, sodium dodecyl sulphate	In vivo pharmacokinetic study of nanosuspension formulations in Sprague Dawley rats illustrated bioavailability enhancement by 1.63 to 2.19 times than crude drug suspension via p.o administration at 14 mg/kg dose	[125]
Media milling techniques	Pluronic, sodium dodecyl sulphate, PVP-K30	The saturation solubility and dissolution rate of DAI was increased through fabrication of nanosuspension. Enhanced cytotoxicity effect was observed in RG2-GBM tumor cells	[27]
Nanocomplexes			
Thermal treatment	Whey protein isolate	Effectively inhibited crystallization, induced 2-fold solubility enhancement and increased DAI stability	[126]
Nanoemulsion			
High-pressure homogenization	Lipoid S100, Tween 80, sodium dodecyl sulfate, Fetal bovine Serum, Ethyl oleate	Cell viability assay using melanoma cell lines (SKMEL30) revealed that nanoemulsion induced significant cell death in comparison to pure DAI (*p* < 0.05) for 48 h of incubation period. However, insignificant (*p* > 0.05) cytotoxic effects were shown by nanoemulsion in human dermal fibroblast (PCS-201-012, normal) cell lines in comparison to DAI solutions and blank formulations for 24 and 48 h of incubation period	[127]
Liposomes			
Ultrasonication and lipid film hydration	Soybean phosphatidylcholine, cholesterol, DSPE-mPEG2000	In vivo pharmacokinetic of liposome in Sprague Dawley rats demonstrated that t_1/2_, MRT_0-t_ and AUC_0-t_ of DAI increased by 1.8-, 1.6- and 2.5-fold in comparison to free DAI	[128]
Self-Micro Emulsifying Drug Delivery System			
Emulsification	Cremophor RH 40, Tween 80, Polyethylene glycol 400	The dissolution rate of SMEDDS was significantly enhanced in contrast to tablets. In vivo pharmacokinetic study in Sprague Dawley rats revealed that AUC_0-12h_ from SMEDDS and DAI suspension (10 mg/kg, p.o.) were 954.32 ± 158.30 ng/mL·min and 380.98 ± 67.59 ng/mL·min, respectively which showed 2.5-fold amplification in bioavailability.	[129]

AUC_0-t_: Area under the plasma concentration-time curve; % CDR: percentage cumulative drug release; DSPE-mPEG2000: PEGylated derivative of 1,2-distearoyl-sn-glycero-3-phosphoethanolamine; MRT_0-t_: mean residence time; PVP-K30: Polyvinylpyrrolidone K-30; PEG 600: Polyethylene glycol 600; SLN: solid lipid nanoparticles; NLCs: nanostructured lipid carriers; PLGA: Poly (lactic-co-glycolic); t_1/2_: half-life_,_ TPGS: Tocopherol Polyethylene Glycol Succinate.

**Table 4:** The state-of-the-art about clinical trial status related to DAI and its therapeutic applications.

Study Tile	Sponsor	Condition	Study Type/Allocation/Intervention Model	NCT no.
Whole soy and DAI on reduction of blood pressure in postmenopausal Chinese women	Chinese University of Hong Kong	Hypertension	Interventional/Randomized/Parallel assignment	01270737
Effects of soy isoflavones on menopausal hot flashes	Beth Israel Deaconess Medical Center	Menopausal symptoms	Interventional/Randomized/Parallel assignment	00179556
Effect of two different isoflavone supplement preparations on gene-expression in postmenopausal women (ISOII)	Wageningen University	Post menopause	Interventional/Randomized/Crossover assignment	01556737
The effects of soy isoflavones to improve the metabolism of glucose and lipids	Sun Yat-sen University	Type 2 diabetes mellitus	Interventional/Randomized/Parallel Assignment	00951912

## Data Availability

Not applicable.
